# A Potential of an Anti-HTLV-I gp46 Neutralizing Monoclonal Antibody (LAT-27) for Passive Immunization against Both Horizontal and Mother-to-Child Vertical Infection with Human T Cell Leukemia Virus Type-I

**DOI:** 10.3390/v8020041

**Published:** 2016-02-03

**Authors:** Hideki Fujii, Mamoru Shimizu, Takuya Miyagi, Marie Kunihiro, Reiko Tanaka, Yoshiaki Takahashi, Yuetsu Tanaka

**Affiliations:** 1 Department of Immunology, Graduate School of Medicine, University of the Ryukyus, Uehara 207, Nishihara-cho, Okinawa 903-0215, Japan; miya_skywalker2008@yahoo.co.jp (T.M.); k138751@eve.u-ryukyu.ac.jp (M.K.); reiko_tanaka@s5.dion.ne.jp (R.T.); ytakah3@med.u-ryukyu.ac.jp (Y.T.); yuetsu@s4.dion.ne.jp (Y.T.); 2 IBL (Immuno-Biological Laboratories Co., Ltd.), Naka 1091-1, Fujioka, Gunma 375-0005, Japan; do-shimizu@ibl-japan.co.jp

**Keywords:** HTLV-I, NOG mice, neutralizing monoclonal antibody, envelope gp46, passive immunity

## Abstract

Although the number of human T-cell leukemia virus type-I (HTLV-I)-infected individuals in the world has been estimated at over 10 million, no prophylaxis vaccines against HTLV-I infection are available. In this study, we took a new approach for establishing the basis of protective vaccines against HTLV-I. We show here the potential of a passively administered HTLV-I neutralizing monoclonal antibody of rat origin (LAT-27) that recognizes epitopes consisting of the HTLV-I gp46 amino acids 191–196. LAT-27 completely blocked HTLV-I infection *in vitro* at a minimum concentration of 5 μg/mL. Neonatal rats born to mother rats pre-infused with LAT-27 were shown to have acquired a large quantity of LAT-27, and these newborns showed complete resistance against intraperitoneal infection with HTLV-I. On the other hand, when humanized immunodeficient mice were pre-infused intravenously with humanized LAT-27 (hu-LAT-27), all the mice completely resisted HTLV-I infection. These results indicate that hu-LAT-27 may have a potential for passive immunization against both horizontal and mother-to-child vertical infection with HTLV-I.

## 1. Introduction

Human T-cell leukemia virus type I (HTLV-I) [1,2] causes both neoplastic and inflammatory diseases, including adult T-cell leukemia (ATL) [3,4] and HTLV-I-associated myelopathy/tropical spastic paraparesis (HAM/TSP) [5,6]. The number of HTLV-I-infected individuals in the world has been estimated at over 10 million [7]. However, no prophylaxis vaccines or drugs against HTLV-I infection are available. HTLV-I is transmitted through contact with bodily fluids containing infected cells, most often from mother to child through breast milk or via blood transfusion. It was demonstrated that HTLV-I efficiently spreads from cell-to-cell via virological synapses [8]. The HTLV-I envelope spike consists of two glycoproteins, cell surface gp46 and trans-membrane gp21 [9], both of which are essential for HTLV-I entry into cells [10].

Since there are little or no genetic mutations in these envelope antigens among HTLV-I strains [11], it is clear that these antigens are the right targets for prophylactic vaccines. Accordingly, a line of evidence showed that a recombinant vaccinia virus (RVV) expressing gp46 and synthetic peptides corresponding to several regions of gp46 conferred immunity against HTLV-I challenge, showing a possibility of active vaccination [12,13,14,15]. However, there are a lot of hurdles before the invention of safe and effective active vaccines. As it has been demonstrated that humanized or human antibodies are safe and effective in various areas of medicine, passive immunization of anti-HTLV-I gp46 neutralizing antibodies may provide a choice for prevention of the spread of HTLV-I. Although antibodies against gp46 antigen and their neutralizing capacity are commonly demonstrated in the sera of HTLV-I-infected individuals, little is known about whether these polyclonal anti-gp46 antibodies can control human-to-human infection of HTLV-I [16]. On the way to establishing a basis for vaccine development against HTLV-I, we previously reported that our anti-gp46 neutralizing mAb (LAT-27) was capable of blocking of HTLV-I infection by direct neutralization and eradicating HTLV-I-infected cells via antibody-dependent-cellular-cytotoxicity (ADCC) *in vitro* [17]. Recently, we showed that LAT-27 is also capable of blocking primary HTLV-I infection in a humanized mouse model [18].

Here, we show that maternally transferred LAT-27 is capable of protecting newborn rats against HTLV-I infection, and suggest that humanized LAT-27 is able to block horizontal infection of humanized mice with HTLV-I. Therefore, humanized LAT-27 may be one of the candidates for passive vaccines against HTLV-I.

## 2. Materials and Methods

### 2.1. Reagents

The medium used throughout was RPMI 1640 medium (Sigma-Aldrich Inc., St. Louis, MO, USA) supplemented with 10% fetal calf serum (FCS), 100 U/mL penicillin and 100 µg/mL streptomycin (hereafter called RPMI medium). Rat and mouse monoclonal antibodies (mAbs) were purified in our laboratory from ascites fluids of CB.17-SCID mice carrying the appropriate hybridomas as described previously [17]. These antibodies were rat IgG2b mAbs anti-gp46 (clones LAT-27), rat IgG2b anti-HIV-1 p24 (clone WAP-24), mouse IgG3 anti-HTLV-I Tax (clone Lt-4). mAbs were labeled with HiLyte Fluor™ 647 using commercial labeling kits (Dojindo, Kumamoto, Japan) according to the manufacturer’s instructions. PE-labeled mouse mAbs against human CD4 were purchased from BioLegend (Tokyo, Japan). Humanized-LAT-27 (hu-LAT-27) and human-mouse chimeric antibody consisting of human IgG1 Fc and a part of mouse anti-CEA were generated in collaboration with IBL (Gunma, Japan) and the information of hu-LAT-27 will be reported elsewhere.

### 2.2. Cell Culture and Syncytium Inhibition Assay

The IL-2-dependent CD4^−^CD8^+^ ILT-M1 cell line derived from a HAM patient was used as a source of HTLV-I (kindly provided by Kannagi of Tokyo medical and dental university) [17]. These cells were maintained in culture using RPMI medium containing 20 U/mL IL-2. Syncytium inhibition assay was carried out using a combination of ILT-M1 and HTLV-I negative Jurkat T-cell lines as reported previously [17]. ILT-M1 cell line was used because of its superiority in inducing syncytia. Briefly, a volume of 25 μL ILT-M1 cell suspension at 2 × 10^6^ cells/mL in 20 U/mL IL-2 containing RPMI media was mixed with 50 μL of serially diluted antibody in a flat-bottom 96-well micro-titer plate for 5 min followed by the addition of a volume of 25 μL Jurkat cell suspension at 2 × 10^6^ cells/mL. After cultivation for 16 h at 37 °C in a 5% CO_2_ humidified incubator, syncytium formation was microscopically observed using an inverted microscope and the concentration of antibody that showed complete blocking of syncytium formation was determined.

### 2.3. ELISA

ELISA was used to quantitate rat and humanized LAT-27 in sera of rats and NOD-SCID/γc null (NOG) mice, respectively. Briefly, HTLV-I gp46 synthetic peptide [19] was coated onto 96-well ELISA plates (Nunc) as an antigen, and the bindings of rat and humanized LAT-27 were detected with HRP-labeled anti-rat and human IgG, respectively.

### 2.4. Animal Experiments

This research was approved by the institutional review boards of the authors’ institutions and written informed consent was obtained from all individuals for the collection of samples and subsequent analysis. The protocols for the use of human PBMCs and animals were approved by the Institutional Review Board and the Institutional Animal Care and Use Committee on clinical and animal research of the University of the Ryukyus prior to initiation of the study. Strains of WKA/H, F344, SD rats were purchased from SLC (Shizuoka, Japan). NOG mice were purchased from the Central Institute of Experimental Animals (Kanagawa, Japan) and were kept in the specific-pathogen-free animal facilities of the Laboratory Animal Center, University of the Ryukyus. Mice were six to seven weeks old at the time of the intra-splenic transplantation of human PBMCs [20]. Fresh PBMCs were isolated from HTLV-I-negative normal donors by a Histopaque-1077 (Sigma) density gradient centrifugation.

### 2.5. Isolation of Human T-Cells from Mouse Spleen

Human CD4^+^ T-cells were isolated from mouse spleen cells by positive immunoselection with the Dynal^®^ CD4-positive isolation kit (Invitrogen), according to the manufacturer’s protocol. In brief, mouse spleen cells were incubated with anti-CD4-coated beads for 30 min at 4 °C under gentle tilt rotation. Captured CD4^+^ T-cells were collected with a magnet (Dynal MPC-S) and detached from beads with DETACHaBEAD CD4/CD8^®^ (Invitrogen). Purity was >99% CD4^+^ T-cells as determined by flow cytometry.

### 2.6. Genomic DNA Extraction and Quantification of HTLV-I Proviral Load

Genomic DNA was extracted by QIAamp kit (QIAGEN, Tokyo, Japan) according to the manufacturer’s instructions. To examine the HTLV-I PVL, we carried out a quantitative PCR method using StepOnePlus (Applied Biosystems) with 100 ng of genomic DNA (roughly equivalent to 10^4^ cells) from PBMC samples as reported previously [21]. Based on the standard curve created by four known concentrations of template, the concentration of unknown samples was determined. Using β-actin as an internal control, the amount of HTLV-I proviral DNA was calculated by the following formula: copy number of HTLV-I tax per 1 × 10^4^ PBMCs = [(copy number of tax) / (copy number of β-actin / 2)] × 10^4^. All samples were performed in triplicate.

### 2.7. Flow Cytometry

Before staining, live cells were Fc-blocked with 2 mg/mL pooled normal human IgG in FACS buffer (PBS containing 0.2% bovine serum albumin and 0.1% sodium azide) for 10 min on ice, and then incubated for 15 min at room temperature. After washing with FACS buffer, the cells were fixed in PBS containing 4% paraformaldehyde (Sigma) for 20 min at room temperature followed by permeabilization and washing in 0.5% saponin + 1% BSA (Sigma) containing FACS buffer. The cells were incubated with 0.1 μg/mL of HiLyte Fluor^®^ 647-labeled anti-Tax mAb (clone Lt-4) for 20 min. Negative control cells were stained with HiLyte Fluor^®^ 647 Lt-4 in the presence of 50 μg/mL of unlabelled Lt-4. Finally, the cells were washed twice and analyzed by standard flow cytometry using a FACSCalibur™ flow cytometer (BD) and FlowJo software (Tree Star).

## 3. Results

### 3.1. Mother-to-Child Transfer of Passively Immunized LAT-27

It has been demonstrated that rats are relatively permissive for *in vivo* infection with HTLV-I. Although rat T-cells transformed with HTLV-I produce no or little infectious HTLV-I virion, rats can be used to evaluate the efficacy of prophylactic vaccines. Thus, we examined whether LAT-27 could be transferred from mother to neonates without loss of protecting capacity against HTLV-I infection. Our preliminary studies showed that spleen cells of SD strain rats were more sensitive to HTLV-I infection *in vitro* than those of WKA/H and F344 strain rats. Thus, we chose SD rats for this *in vivo* study.

At first, pregnant SD rats were infused i.p. with 25 mg/head of either LAT-27 or isotype control mAb two times on –7 d and –2 d of delivery (*n* = 2). Two days after the birth, blood samples were collected from both mothers and newborns, and LAT-27 concentrations of their sera were quantitated using ELISA (Table 1).

**Table 1:** Mother-to-offspring transfer of LAT-27 in rats. Two pregnant SD rats were infused i.p. with 25 mg/head LAT-27 two times on −7 d and −2 d delivery. Two days after delivery, LAT-27 concentration of each serum was quantitated by ELISA.

Pregnant Rat #1	Serum LAT-27 conc. (μg/mL)		Pregnant Rat #2	Serum LAT-27 conc. (μg/mL)
Mother	102		Mother	51
Offspring-1	204		Offspring-1	102
Offspring-2	204		Offspring-2	51
Offspring-3	102		Offspring-3	102
Offspring-4	102		Offspring-4	102
Offspring-5	204		Offspring-5	51
Offspring-6	102		Offspring-6	51
Offspring-7	204		Offspring-7	51
Offspring-8	204		Offspring-8	102
Offspring-9	102		Offspring-9	102
			Offspring-10	51

**Figure 1:**
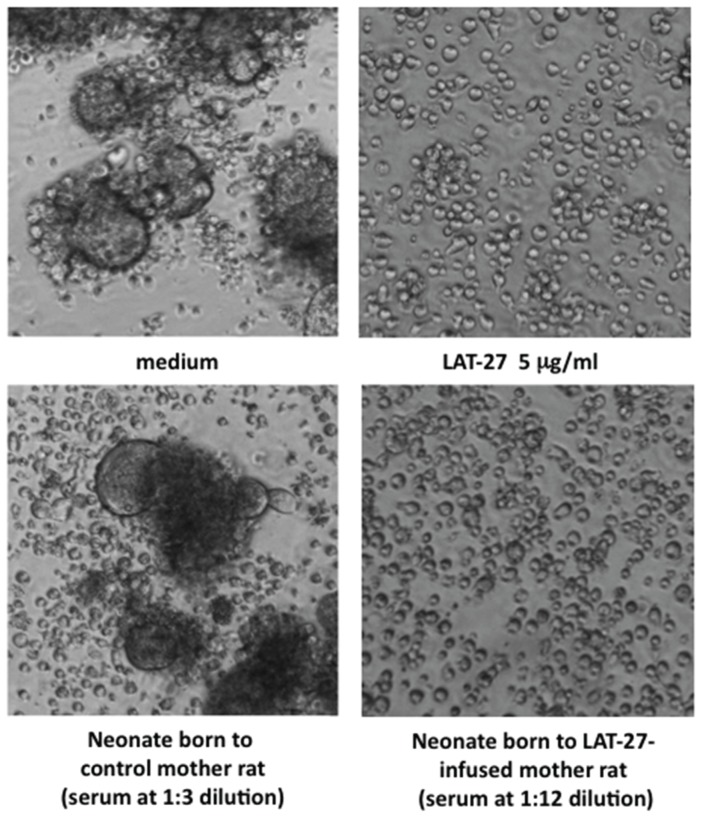
Neutralization of HTLV-I by mother-to-offspring-transferred LAT-27. Syncytium inhibition assay was carried out as described in Materials and Methods. ILT-M1 cells were treated with diluted serum or purified antibody in a flat-bottom 96-well micro-titer plate for 5 min, and then co-cultured with Jurkat cells. After cultivation for 16 h, syncytium formation was microscopically observed using an inverted microscope at magnification of 200×.

The data showed that a substantial dose of LAT-27 was transferred to the neonates from the mothers. It is of interest that the LAT-27 concentrations in newborn rat sera were equivalent to those in mother sera, suggesting an efficient transfer of LAT-27.

In order to confirm that the transferred LAT-27 in neonates retained neutralizing activity, we performed the syncytium inhibition assay (Figure 1).

Overnight co-culture of the HTLV-I-producing ILT-M1 cells and HTLV-I-negative Jurkat T-cells in media alone gave rise to the generation of a number of syncytia (Figure 1 upper left panel), which was completely inhibited by the addition of LAT-27 (5 μg/mL) (Figure 1 upper left panel). In contrast to that, the control serum obtained from a neonate born to an untreated normal mother did not inhibit the syncytium formation even at 1:3 dilution, thus the serum from a LAT-27-adminstered mother did neutralize HTLV-I infection at 1:12 dilutions. Taken together, these data indicated that LAT-27 could be transferred from mother to offspring without any loss of its neutralization property.

### 3.2. LAT-27 from Mothers Protects Newborns against HTLV-I Infection

**Figure 2:**
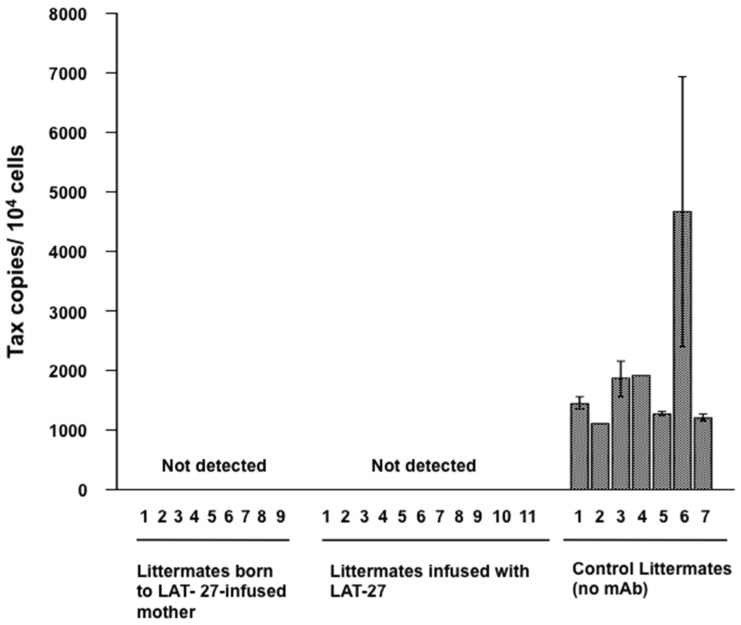
Protection of newborns from HTLV-I infection by mother-to-child-transferred LAT-27. Nine littermates from LAT-27 passively immunized mother rat (**left**), 11 littermates directly injected with LAT-27 (**center**) before infection, and seven naïve littermates (**right**) were i.p. injected with MMC-treated ILT-M1 cells. After three weeks, rats were tested for HTLV-I infection.

Then, we addressed whether mother-to-child-transferred LAT-27 could protect newborn rats against HTLV-I infection. Pregnant SD rats received 25 mg/head LAT-27 two days before delivery, and their newborns were challenged i.p. with the mitomycin C-treated HTLV-I-producing cells on day 1 after birth. As controls, two newborn littermates of non-antibody-administered mothers were infected with HTLV-I before or after administration with 2 mg/head LAT-27. Three weeks after infection, rats were sacrificed and spleen cells were subjected to quantitative real-time PCR analysis for testing HTLV-I infection. Figure 2 showed that the rats born to the LAT-27-treated mother rat as well as the newborn rats directly administered LAT-27 after birth were negative for proviral DNA, suggesting that mother-to-child-transferred LAT-27 could work *in vivo*.

### 3.3. Retention of Humanized LAT-27 in NOG Mice

**Figure 3:**
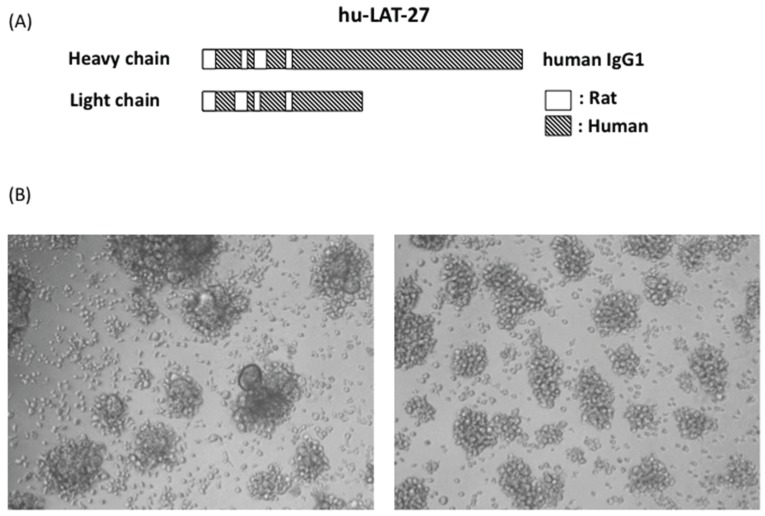
Schematic illustration of humanized LAT-27 and its neutralizing activity. Syncytium inhibition assay was carried out with either 200 μg/mL of normal human IgG (**left** of **B**) and 5 μg/mL of humanized LAT-27. After cultivation for 24 h, syncytium formation was microscopically observed using an inverted microscope at magnification of 100× (**right** of **B**).

For future prophylactic and clinical utilization, we have succeeded in generating humanized LAT-27 (hu-LAT-27) as shown in Figure 3. hu-LAT-27 consists of a complementarity-determining region (CDR) of the original rat LAT-27 and human IgG1 backbone as illustrated in Figure 3A.

hu-LAT-27 was produced in CHO cells. Due to a patent matter, the gene sequence of hu-LAT-27 will be reported elsewhere. The *in vitro* neutralization test showed that the minimum concentration of hu-LAT-27 for complete inhibition of syncytium formation was 5 μg/mL, which was comparable to the original rat LAT-27 (Figure 3B), demonstrating no decay in neutralizing ability after humanization. In order to examine whether hu-LAT-27 could work *in vivo*, we firstly tested the retention time of hu-LAT-27 in NOG mice. Mice were injected i.v. with 1 mg/head hu-LAT-27 (*n* = 3). Sera were collected daily for five days and the concentration of hu-LAT-27 was quantitated by ELISA as described in the Materials and Methods. The data presented in Table 2 indicated that serum levels of hu-LAT-27 gradually decreased day by day. The half-life of administrated LAT-27 was estimated to be approximately two days.

**Table 2:** Retention of humanized LAT-27 in NOG mice. Each of three NOG mice was infused i.v. with 1 mg/head hu-LAT-27, and the LAT-27 concentrations in their sera were measured daily by ELISA using gp46 peptide (180–204) as antigen and secondary anti-human IgG-HRP, in which purified hu-LAT-27 was used to draw a standard curve.

Days after Injection	hu-LAT-27 Concentration in Serum (μg/mL)		
	NOG#1	NOG#2	NOG#3
1	268	249	277
2	114	155	124
3	79	88	80
4	57	86	61
5	49	47	48

### 3.4. Protection of hu-PBL-NOG Mice against HTLV-I Infection by hu-LAT-27

**Figure 4:**
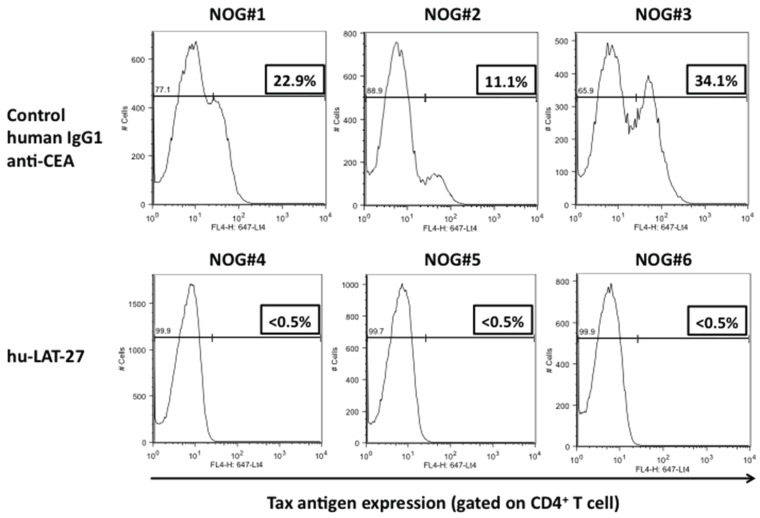
Protection of hu-PBL-NOG mice against HTLV-I by hu-LAT-27. The Mitomycin C (MMC)-treated HTLV-I-infected ILT-M1 (1 × 10^6^ cells) was mixed with freshly isolated PBMCs (2 × 10^6^ cells) from healthy donor in a final volume of 50 µL and then were directly injected into the spleens of NOG mice. MAbs (0.5 mg each: lower panels) or control IgG (0.5 mg each: upper panels) was inoculated i.v. one hour before cell transplantation. Fourteen days after cell transplantation, mice were sacrificed, blood was collected by cardiocentesis, and human lymphocytes were recovered from the spleen. The bisector indicated in all panels showed tax negative edge of histogram.

Then, we tested the protective efficacy of hu-LAT-27 mAbs against *in vivo* infection with HTLV-I using our humanized mouse model as reported previously [18]. PBMCs from HTLV-I-negative donors were transplanted directly into the spleen of NOG mice together with the mitomycin-C-treated HTLV-I-producing cells. They were intra-peritoneally infused with either hu-LAT-27 or control human chimeric anti-CEA mAb before HTLV-I infection. Fourteen days after infection, these mice were sacrificed and fresh spleen cells were isolated, cultured for 24 h and tested for HTLV-I infection by flow cytometry. As shown in Figure 4A, human CD4^+^ T-cells from all mice immunized with the hu-LAT-27 before HTLV-I infection were negative for Tax antigen, suggesting complete protection against HTLV-I infection (lower panels). PCR testing of HTLV-I proviral loads supported this conclusion. The protective effect was hu-LAT-27-specific since the control anti-CEA did not confer protection against the infection (upper panels). When hu-LAT-27 was administered one day after HTLV-I infection, it did not protect mice against HTLV-I infection. These results suggested that hu-LAT-27 could be used for prophylaxis against HTLV-I infection *in vivo* when a sufficient amount of it was infused before infection with HTLV-I.

## 4. Discussion

The present study suggested a potential of monoclonal anti-gp46 neutralizing LAT-27 for passive immunization for prophylaxis against HTLV-I infection. The main mechanism for LAT-27-mediated protection of animals against HTLV-I *in vivo* is most likely neutralization via inducing conformational changes in the gp46 structure. In addition, as we have previously reported, LAT-27 may also inhibit HTLV-I infection by eliminating HTLV-I-producing cells via HTLV-1-specific ADCC in the presence of NK cells [17]. In the present study, we have chosen an excess dose of LAT-27 for infusion. Since the minimum HTLV-I neutralizing antibody concentration of LAT-27 *in vitro* is as low as 5 μg/mL, further studies are required to determine the minimum dose of LAT-27 for perfect *in vivo* protection against HTLV-I in each animal system.

Using a rat model, the present study first showed that LAT-27 could be transferred from dams to infant rats, and that the transferred LAT-27 was active for HTLV-I neutralization and protected the infants against intraperitoneal HTLV-I infection. It remains to be determined how long the antibodies were sustained in the littermates born to pregnant rats infused with LAT-27. In rats, it is known that maternal antibodies are transferred equally effectively to infants either via placenta or from the gut by breastfeeding [22]. It should be emphasized that administration of LAT-27 to pregnant rats did not show any obvious effects on delivery and growth of infants, showing the safety of this mAb in rats at least. It remains to be tested whether the transferred LAT-27 is protective against oral HTLV-I infection. Although it has been shown that rats are relatively susceptible to oral infection with HTLV-I [23], our preliminary studies failed to infect newborn (two-day-old) rats with HTLV-I by feeding cow milk serum containing live HTLV-I at least producing cells. In order to establish a good model for oral HTLV-I infection, choices of better rat strain, HTLV-I at least producing cells and timing of infection may be a key. Further studies are in progress.

For future clinical studies in humans, it is necessary to humanize the original LAT-27 (rat origin). In collaborative investigation with IBL Co., Ltd., we have succeeded in the generation of hu-LAT-27, and examined its function *in vitro* and *in vivo*. Similar to rat LAT-27, hu-LAT27 neutralized HTLV-I *in vitro* at a minimum concentration of 5 μg/mL (Figure 1), showing that there is no loss of neutralizing activity after humanization. Furthermore, hu-LAT-27 was capable of protecting NOG mice reconstituted with human PBMCs from intraperitoneal infection with HTLV-I (Figure 4). The half-life of hu-LAT-27 in NOG mice was roughly estimated two days. Since half-life of IgG *in vivo* is dependent on its affinity to neonatal FcR, such a short lifetime of hu-LAT-27 in NOG mice (Table 2) will be prolonged up to three weeks in humans, as in the cases of other humanized mAbs [24].

The present data suggest that hu-LAT-27 mAb will be useful in passive immunization against HTLV-I infection. The rationale of using these monoclonal antibodies instead of polyclonal antibodies obtained from healthy HTLV-I carriers for the prevention of HTLV-I infection is that, in contrast to HIV-1, the genome of HTLV-I is stable [11], and thus the LAT-27 reactive-neutralizing epitope (gp46 amino acid 191–196 of MT-2 virus) is broadly conserved among wild HTLV-I strains. Indeed, LAT-27 reacted all HTLV-I-producing cell lines tested, including naturally infected cell lines from ATL and HAM/TSP patients and asymptomatic carriers. So far, the potential of passive immunization with IgG from HTLV-I carriers has been show in rabbit [25,26], monkey [27] and humanized NOG mouse models [18]. Since polyclonal antibodies contain non-neutralizing antibodies, the efficacy of neutralization by neutralizing antibody could be interfered with by those non-neutralizing anti-gp46 antibodies. More importantly, since the total availability of anti-HTLV-I serum is limited, production capability in a large quantity of Good Manufacturing Practice-grade of HTLV-I neutralizing mAb may be a prerequisite for antibody-based prophylaxis against HTLV-I. Another choice, instead of hu-LAT-27, will be human mAbs with HTLV-I neutralizing capacity, as several human mAbs have been reported [28].

We anticipate that hu-LAT-27 mAb will be used as a safe passive vaccine against both horizontal and vertical infection with HTLV-I for people at high risk of HTLV-I infection, including adults and babies born to HTLV-I carriers. In addition, since organ transplantation to HTLV-I-negative recipients from HTLV-I-positive donors is another risk factor of HTLV-I infection and the development of HAM/TSP [29], pretreatment of recipients with hu-LAT-27 may be useful for the prevention of HTLV-I infection. It may be also possible that, for HTLV-I carriers whose anti-HTLV-I neutralizing antibody titers are low, administration of LAT-27 may function to inhibit further spreading of HTLV-I *in vivo*. Thus, it will be worthy to validate hu-LAT-27 in an animal model in more detail before starting a clinical study. In addition to this passive vaccine, in order to control HTLV-I infection all over the world, it is clear that an active vaccine which can elicit or boost anti-HTLV-I gp46 neutralizing antibody titer should be developed.

## 5. Conclusions

This study describes experimental results indicating a possible concept that passive immunization with our humanized anti-gp46 neutralizing mAb (hu-LAT-27) reduces the chance of HTLV-I infection of adults and babies at high risk. A combination of hu-LAT-27-based passive vaccination and yet-to-be-developed active vaccine might be potent in preventing the spread of HTLV-I infection around the world.
